# Machine learning models for early mortality prediction in severe fever with thrombocytopenia syndrome

**DOI:** 10.1016/j.isci.2026.114843

**Published:** 2026-01-29

**Authors:** Chenxi Zhao, Tingyu Zhang, Ziruo Ge, Ling Lin, Di Tian, Yi Shen, Zhenghua Zhao, Jingxia Wang, Jianming Lai, Yanli Xu, Jianping Duan, Zhihai Chen

**Affiliations:** 1 National Key Laboratory of Intelligent Tracking and Forecasting for infectious Diseases, Beijing Ditan Hospital, Capital Medical University, Beijing 100015, China; 2 Department of Infectious Diseases, Yantai Qishan Hospital, Yantai, Shandong Province 264001, China; 3 Department of Infectious Diseases, Dandong Infectious Disease Hospital, Dandong, China; 4 Department of Infectious Disease, Tai’an City Central Hospital, Qingdao University, Tai’an, Shandong Province 271000, China; 5 Department of Infectious Diseases, The Sixth People’s Hospital of Qingdao, Tai’an, Shandong Province 266033, China

**Keywords:** public health, artificial intelligence applications

## Abstract

Severe fever with thrombocytopenia syndrome (SFTS) is a high-fatality viral disease where early mortality risk prediction is vital for clinical management. This retrospective multicenter cohort study enrolled 1,690 hospitalized SFTS patients from five Chinese hospitals (2014–2023) to develop, validate, and deploy an interpretable machine learning (ML) model for early mortality risk assessment. Using LASSO regression for feature selection then comparing eight ML algorithms, the XGBoost model achieved an AUC of 0.916 in the training cohort and 0.905 in the temporal validation cohort. SHapley Additive exPlanations (SHAP) analysis identified six key predictors, which were used to deploy a real-time, open-access web-based tool (https://sftsprognosis.com) that provides individualized risk predictions with visual explanations. The XGBoost model has the potential to enhance timely clinical decision-making, facilitate efficient allocation of critical care resources, and provide a generalizable framework for applying machine learning in the management of emerging infectious diseases.

## Highlights

• This retrospective multicentre cohort study collected 10 years from five hospitals in China
• Eight machine learning models were trained, validated, and interpreted using SHAP analysis
• An XGBoost model was developed to predict early mortality risk in patients with SFTS
• A publicly accessible, real-time web tool was deployed to facilitate timely decision-making

## Graphical abstract

**Figure:**
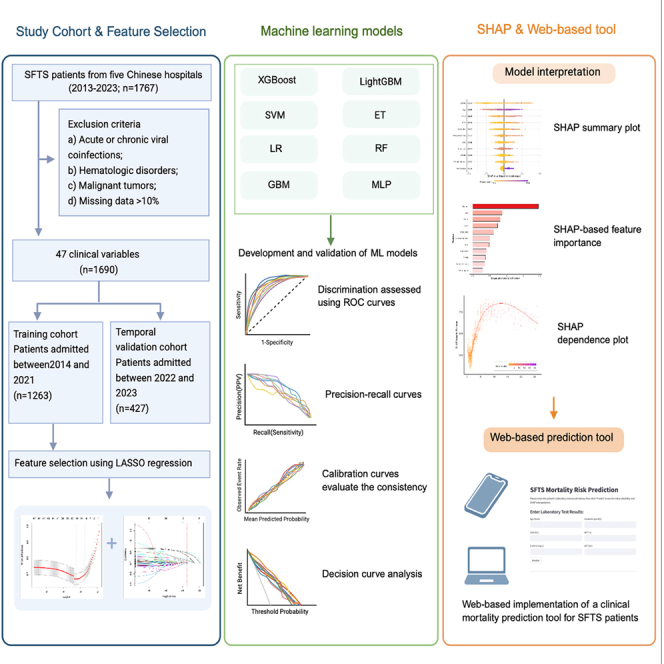

## Introduction

Severe fever with thrombocytopenia syndrome (SFTS) is an emerging, tick-borne infectious disease caused by the SFTS virus (SFTSV), first identified in China in 2009.^1^ In recent years, SFTS has been increasingly recognized as an emerging public health threat, with confirmed cases reported in a number of east and southeast Asian countries, such as South Korea, Japan, Vietnam, Thailand, and Pakistan, among others.^2^^,^^3^^,^^4^^,^^5^^,^^6^ The virus is primarily transmitted through tick bites, parthenogenetic Asian long horned ticks are thought to contribute significantly to the rapid geographical spread of SFTSV.^7^ Additionally, evidence suggests that SFTSV can also be transmitted from human to human, particularly through contact with blood or body fluids of infected individuals.^8^^,^^9^ Major laboratory findings in SFTS patients include thrombocytopenia, leukopenia, elevated liver enzyme levels, and coagulation abnormalities.^10^ Clinically, patients commonly present with fever, gastrointestinal symptoms, hemorrhagic manifestations, disseminated intravascular coagulation (DIC), and multi-organ failure.^11^ Severe cases often progress rapidly and are associated with a high case fatality rate (CFR). According to a recent meta-analysis, the overall pooled CFR of SFTS was 7.80% (95% confidence interval [CI], 7.01%–8.69%),^12^ posing a substantial challenge to clinical management and disease control.

Currently, no vaccines or specific antiviral therapies have been approved for the treatment of SFTS. Although the efficacy of ribavirin has been suggested in a limited number of clinical studies;^13^ favipiravir has shown significant therapeutic effects only in patients with low viral load, younger age, and shorter duration of illness.^14^ As a result, supportive care remains the cornerstone of clinical management.^15^ Therefore, early risk identification and stratification are critical for timely therapeutic interventions and improving clinical outcomes. However, in clinical practice, there remains a lack of efficient and generalizable tools for predicting mortality risk in patients with SFTS. Previous studies have identified several potential prognostic factors associated with fatal outcomes, including advanced age, impaired consciousness, coagulopathy, liver dysfunction, and pronounced cytokine abnormalities.^16^^,^^17^^,^^18^ Although several models have been developed to predict mortality and disease severity using logistic regression or clinical scoring approaches, most of these models are limited by small sample sizes, single-center study designs, lack of external validation, and limited clinical applicability.

With the advancement of machine learning (ML) techniques in clinical medicine, data-driven predictive models offer promising potential to capture complex variable interactions between clinical features and outcomes.^19^ Recent studies have explored the application of ML algorithms to improve predictive performance in both clinical and basic research settings, including the prediction of secondary infections in COVID-19 and the identification of genetic factors influencing virus receptor binding.^20^^,^^21^ Chen et al. developed an interpretable model to predict ICU readmission in acute pancreatitis using synthetic minority over-sampling technique (SMOTE), ML, and SHapley Additive exPlanations (SHAP) analysis.^22^ Similarly, Sun et al. developed an interpretable ML model for predicting 28-day mortality in ICU patients with diabetes and atrial fibrillation, highlighting the clinical potential of ML-based triage tools in high-risk populations.^23^ Existing clinical scoring systems or laboratory markers for SFTS lack sufficient generalizability and clinical utility in real-world settings. While ML models have shown promise in risk prediction across various diseases, few have been effectively translated into accessible tools to support frontline clinical decision-making. In the context of SFTS, where disease progression can be rapid and unpredictable, a reliable and readily available online prediction tool could facilitate early identification of high-risk patients and inform timely intervention and resource allocation. In this study, we aimed to develop and validate an ML-based mortality risk prediction model using a large, multicenter clinical cohort from five hospitals in China over a ten-year period, and to deploy it as a publicly accessible web-based application for real-time clinical use.

## Results

### Comparison of baseline characteristics between training and validation cohorts

A total of 1,690 patients with confirmed SFTS were included, comprising 1,378 survivors and 312 non-survivors, an overall CFR of 18.46%. The training cohort consisted of 1,263 patients admitted between January 2014 and December 2021, while the temporal validation cohort included 427 patients admitted between January 2022 and December 2023. Baseline demographic, clinical, and laboratory characteristics of the study population are presented in Table 1.

**Table 1:** Baseline characteristics of patients with SFTS in the training and validation cohorts

	Total (*n* = 1,690)	Training cohort (*n* = 1,263)			Validation cohort (*n* = 427)		
		Survivors (*n* = 1,026)	Non-survivors (*n* = 237)	*P*^*a*^	Survivors (*n* = 352)	Non-survivors (*n* = 75)	*P*^*b*^
Sex (male), n (%)	826 (48.9)	488 (47.6)	124 (52.3)	0.212	174 (49.4)	40 (53.3)	0.627
Age (years)	65.0 (56.0–72.0)	63.0 (55.0–71.0)	70.0 (64.0–76.0)	<0.001	63.0 (55.0–71.0)	69.0 (64.0–75.5)	<0.001
Peak temperature (°C)	38.8 (38.2–39.0)	38.80 (38.10–39.00)	38.60 (38.40–39.00)	0.999	38.90 (38.30–39.00)	38.50 (38.00–39.00)	0.008

**Signs and symptoms (n,%)**							

Chill	648 (38.3)	409 (39.9)	72 (30.4)	0.008	138 (39.2)	29 (38.7)	1.000
Weakness	1,263 (74.7)	771 (75.1)	167 (70.5)	0.160	272 (77.3)	53 (70.7)	0.285
Fatigue	858 (50.8)	518 (50.5)	124 (52.3)	0.662	179 (50.9)	37 (49.3)	0.911
Headache	335 (19.8)	217 (21.2)	29 (12.2)	0.002	80 (22.7)	9 (12.0)	0.055
Myalgia	628 (37.2)	381 (37.1)	84 (35.4)	0.680	140 (39.8)	23 (30.7)	0.179
Arthralgia	335 (19.8)	214 (20.9)	45 (19.0)	0.580	71 (20.2)	5 (6.7)	0.009
Inappetence	1,279 (75.7)	771 (75.1)	172 (72.6)	0.461	283 (80.4)	53 (70.7)	0.087
Nausea	732 (43.3)	470 (45.8)	74 (31.2)	<0.001	157 (44.6)	31 (41.3)	0.697
Vomiting	406 (24.0)	255 (24.9)	41 (17.3)	0.017	91 (25.9)	19 (25.3)	1.000
Diarrhea	408 (24.1)	241 (23.5)	63 (26.6)	0.358	86 (24.4)	18 (24.0)	1.000
Lymphadenectasis	271 (16.0)	156 (15.2)	44 (18.6)	0.239	60 (17.0)	11 (14.7)	0.740
Neurological abnormalities	344 (20.4)	164 (16.0)	92 (38.8)	<0.001	60 (17.0)	28 (37.3)	<0.001

**Laboratory test (median and IQR)**							

WBC (10ˆ9/L)	2.70 (1.67–4.60)	2.73 (1.71–4.57)	2.53 (1.64–4.81)	0.484	2.92 (1.71–4.60)	2.19 (1.49–4.37)	0.097
Neutrophils (10ˆ9/L)	1.56 (0.91–2.93)	1.51 (0.90–2.88)	1.69 (1.00–3.34)	0.067	1.56 (0.90–2.89)	1.78 (1.04–3.38)	0.334
Lymphocytes (10ˆ9/L)	0.66 (0.40–1.18)	0.70 (0.42–1.21)	0.50 (0.32–0.84)	<0.001	0.76 (0.44–1.31)	0.41 (0.30–0.80)	<0.001
Monocytes (10ˆ9/L)	0.18 (0.09–0.44)	0.19 (0.10–0.42)	0.13 (0.06–0.40)	0.002	0.23 (0.10, 0.51)	0.11 (0.05–0.35)	<0.001
Eosinophils (10ˆ9/L)	0.00 (0.00–0.01)	0.00 (0.00–0.01)	0.00 (0.00–0.01)	0.604	0.00 (0.00–0.01)	0.00 (0.00–0.01)	0.326
Basophils (10ˆ9/L)	0.01 (0.00–0.02)	0.01 (0.00–0.02)	0.00 (0.00–0.02)	0.166	0.01 (0.00–0.02)	0.00 (0.00–0.01)	0.189
RBC (10ˆ12/L)	4.50 (4.16–4.87)	4.51 (4.17–4.86)	4.53 (4.07–4.96)	0.696	4.47 (4.16–4.86)	4.52 (4.00–4.88)	0.684
Hemoglobin (g/L)	137.0 (126.0–148.0)	136.5 (127.0–147.0)	139.0 (124.0–153.0)	0.085	136.0 (126.0–149.0)	137.0 (129.0–152.0)	0.432
Platelet (10ˆ9/L)	56.00 (40.00–76.00)	60.00 (42.00–81.00)	48.00 (34.00–66.00)	<0.001	56.00 (45.75–75.00)	40.00 (30.00–61.50)	<0.001
MPV (fL)	11.00 (10.20–11.80)	11.00 (10.20–11.90)	10.90 (10.00–11.70)	0.241	11.00 (10.30–11.90)	10.50 (9.75–11.75)	0.019
Serum potassium (mmol/L)	3.78 (3.43–4.10)	3.72 (3.40–4.07)	3.97 (3.60–4.50)	<0.001	3.73 (3.40–4.03)	3.94 (3.60–4.40)	0.002
serum sodium (mmol/L)	134.95 (131.30–138.00)	134.70 (131.50–137.80)	135.00 (131.00–138.00)	0.516	135.00 (131.38–137.48)	134.00 (130.90–138.00)	0.561
BUN (mmol/L)	5.60 (3.92–7.35)	5.20 (3.80–6.64)	7.66 (5.69–12.60)	<0.001	5.30 (3.75–6.86)	6.29 (4.29–8.49)	<0.001
Creatinine (umol/L)	65.00 (52.00–82.27)	62.65 (52.00–78.10)	85.00 (65.00–128.00)	<0.001	62.00 (52.00–76.55)	76.00 (63.05–85.00)	<0.001
LDH (U/L)	646.00 (389.00–941.91)	575.00 (365.09–900.00)	917.00 (742.00–1,695.00)	<0.001	624.67 (377.75–901.85)	900.00 (616.00–1,560.25)	<0.001
CK (U/L)	403.50 (169.25–1,047.50)	322.00 (144.93–797.75)	1118.00 (400.00–2,000.00)	<0.001	360.00 (146.50–889.25)	786.00 (362.75–1,611.17)	<0.001
CK-MB (U/L)	16.59 (8.00–30.00)	15.03 (7.34–26.00)	24.00 (11.17–57.00)	<0.001	15.00 (7.13–28.00)	26.00 (15.00–61.50)	<0.001
CRP (mg/L)	3.29 (0.80–10.00)	2.58 (0.30–8.31)	7.60 (5.40–17.93)	<0.001	2.66 (0.72–7.21)	7.60 (4.11–13.59)	<0.001
PT (s)	12.40 (11.50–13.00)	12.40 (11.50–12.90)	12.90 (11.90–13.80)	<0.001	12.30 (11.50–12.80)	12.70 (11.70–13.40)	0.002
APTT (s)	41.95 (37.40–50.70)	41.20 (36.60–47.80)	53.50 (45.50–69.90)	<0.001	41.20 (35.40–47.15)	54.60 (46.40–63.45)	<0.001
TT (s)	19.70 (17.80–23.10)	19.30 (17.70–22.10)	23.10 (19.30–29.20)	<0.001	19.00 (17.60–21.60)	22.70 (18.80–27.10)	<0.001
D-dimer (ug/ml)	2.10 (1.15–4.01)	1.70 (1.13–3.00)	5.43 (3.22–8.91)	<0.001	1.80 (1.10–3.06)	7.00 (4.00–9.00)	<0.001
ALT (U/L)	72.85 (42.44–136.58)	69.10 (39.00–123.00)	89.80 (54.70–201.50)	<0.001	73.69 (41.88–137.53)	87.00 (57.70–182.50)	0.032
AST (U/L)	134.45 (67.78–289.67)	117.00 (58.05–237.50)	285.90 (115.30–736.00)	<0.001	135.10 (63.52–270.83)	267.00 (149.40–494.70)	<0.001
TBiL (umol/L)	9.90 (7.40–13.47)	9.72 (7.22–13.29)	10.70 (8.10–16.00)	0.001	9.90 (7.71–13.00)	10.09 (7.68–12.85)	0.665
DBiL (umol/L)	4.00 (2.77–5.86)	3.76 (2.60–5.50)	5.10 (3.66–8.57)	<0.001	3.90 (2.77–5.40)	5.18 (3.35–7.65)	<0.001
Albumin (g/L)	32.00 ± 4.62	32.5 ± 4.49	30.3 ± 4.81	<0.001	32.21 ± 4.59	30.08 ± 4.28	<0.001
Globulin (g/L)	25.70 (22.60–28.80)	25.61 (22.60–28.76)	26.00 (23.19–28.58)	0.377	25.54 (22.60–28.95)	25.80 (22.05–29.34)	0.908
GGT (U/L)	34.00 (20.70–73.00)	33.00 (20.00–68.00)	42.30 (25.00–113.00)	<0.001	32.00 (19.00–64.25)	41.24 (25.00–96.50)	0.010
Cholinesterase (U/L)	5,805.47 (4,641.25–6,899.41)	5,909.00 (4,705.00–7,060.52)	5,424.00 (4,579.40–6,103.96)	<0.001	5,730.00 (4,633.75–6,939.00)	5,529.70 (4,554.65–6,601.98)	0.182
ALP (U/L)	63.00 (51.26–82.30)	62.30 (51.27–79.97)	69.80 (54.00–116.60)	<0.001	60.35 (50.00–78.25)	65.00 (52.00–92.50)	0.083
TBA (umol/L)	5.40 (3.40–10.50)	4.60 (3.12–9.20)	9.00 (7.10–18.70)	<0.001	4.50 (3.00–8.20)	9.20 (6.35–22.25)	<0.001

*P*^a^, comparison between survivors and non-survivors in the training cohort. *P*^b^, comparison between survivors and non-survivors in the validation cohort. Categorical variables were presented as frequencies and percentages (n, %) and compared using the Chi-square test. Continuous variables with a normal distribution were expressed as mean ± standard deviation (SD) and compared using the independent samples *t* test. Non-normally distributed continuous variables were reported as median and interquartile range (IQR) and compared using the Mann-Whitney U test. WBC, white blood cell; RBC, red blood cell; MPV, mean platelet volume; BUN, blood urea nitrogen; LDH, lactate dehydrogenase; CK, creatine kinase; CK-MB, creatine kinase-MB; CRP, C-reactive protein; PT, prothrombin time; APTT, activated partial thromboplastin time; TT, thrombin time; ALT, alanine aminotransferase; AST, aspartate aminotransferase; TBiL, total bilirubin; DBiL, direct bilirubin; GGT, gamma-glutamyl transferase; ALP, alkaline phosphatase; TBA, total bile acid. Race/ancestry/ethnicity information was not systematically collected in this cohort. All participants were recruited from hospitals in mainland China.

In terms of demographics, 48.9% of the overall study population were male. No significant differences in sex distribution were observed between survivors and non-survivors in either the training cohort (*p* = 0.212) or the validation cohort (*p* = 0.627). However, non-survivors were significantly older than survivors in both the training cohort (*p* < 0.001) and the validation cohort (*p* < 0.001) (Table 1). Among clinical symptoms, the most frequently reported were inappetence (75.7%), weakness (74.7%), fatigue (50.8%), nausea (43.3%), chills (38.3%), and myalgia (37.2%). Neurological abnormalities were significantly more common in non-survivors compared to survivors in both cohorts (*p* < 0.001). Non-survivors exhibited significantly lower levels of PLT (*p* < 0.001, *p* < 0.001), lymphocyte count (*p* < 0.001, *p* < 0.001), monocyte count (*p* = 0.002, *p* < 0.001), and albumin (*p* < 0.001, *p* < 0.001), along with higher levels of D-dimer (*p* < 0.001, *p* < 0.001), C-reactive protein (CRP) (*p* < 0.001, *p* < 0.001), alanine aminotransferase (ALT) (*p* < 0.001, *p* = 0.032), aspartate aminotransferase (AST) (*p* < 0.001, *p* < 0.001), lactate dehydrogenase (LDH) (*p* < 0.001, *p* < 0.001), serum potassium (*p* < 0.001, *p* = 0.002), blood urea nitrogen (BUN) (*p* < 0.001, *p* < 0.001), creatinine (*p* < 0.001, *p* < 0.001), prothrombin time (PT) (*p* < 0.001, *p* = 0.002), activated partial thromboplastin time (APTT) (*p* < 0.001, *p* < 0.001), thrombin time (TT) (*p* < 0.001, *p* < 0.001), gamma-glutamyl transferase (GGT) (*p* < 0.001, *p* = 0.010), total bile acid (TBA) (*p* < 0.001, *p* = 0.007) compared with survivors in both the training and validation cohorts, respectively. No significant difference was observed in white blood cell count, neutrophil count, eosinophil count, red blood cell count, hemoglobin, or globulin levels between survivors and non-survivors in either the training or validation cohorts (*p* > 0.05) (Table 1).

### Feature selection using LASSO regression

**Figure 1:**
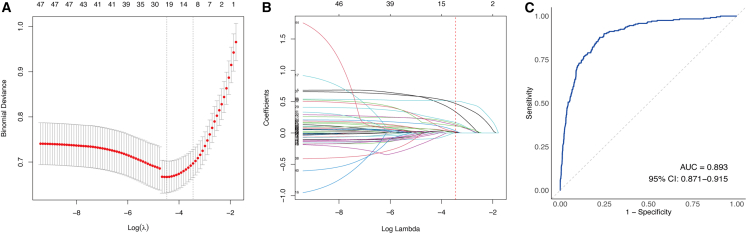
LASSO regression-based feature selection and model performance (A) LASSO cross-validation curve. 10-fold cross-validation was performed to determine the optimal penalty parameter (λ). Points represent the mean binomial deviance across cross-validation folds, and error bars indicate ±1 standard error (SE). The left dashed line represents λ.min, and the right dashed line represents λ.1SE, where 11 predictors were selected. (B) LASSO coefficient profiles of the 47 candidate predictors. The red dashed line indicates the selected value of λ.1SE. (C) ROC curve of the LASSO model in the training cohort.

A total of 47 candidate clinical and laboratory variables as input features in the training cohort. Feature selection was performed using LASSO regression with 10-fold cross-validation, applying the λ.1SE criterion to optimize model parsimony. Using a fixed random seed, 11 variables with nonzero coefficients were retained, including age, neurological abnormalities, K^+^, creatinine, LDH, CK, CKMB, PT, APTT, D-dimer, and AST (Figures 1A and 1B). The predictive performance of the LASSO-derived model was assessed by ROC curve analysis, with an area under the curve (AUC) of 0.893 (95% CI, 0.871–0.915) (Figure 1C).

Given that 237 adverse events were observed among 1,263 patients with SFTS, and 11 variables were selected for model development, the resulting events per variable was 21.545, indicating a low risk of overfitting and supporting the model’s robustness and generalizability.

### Comparative performance of eight machine learning models

**Figure 2:**
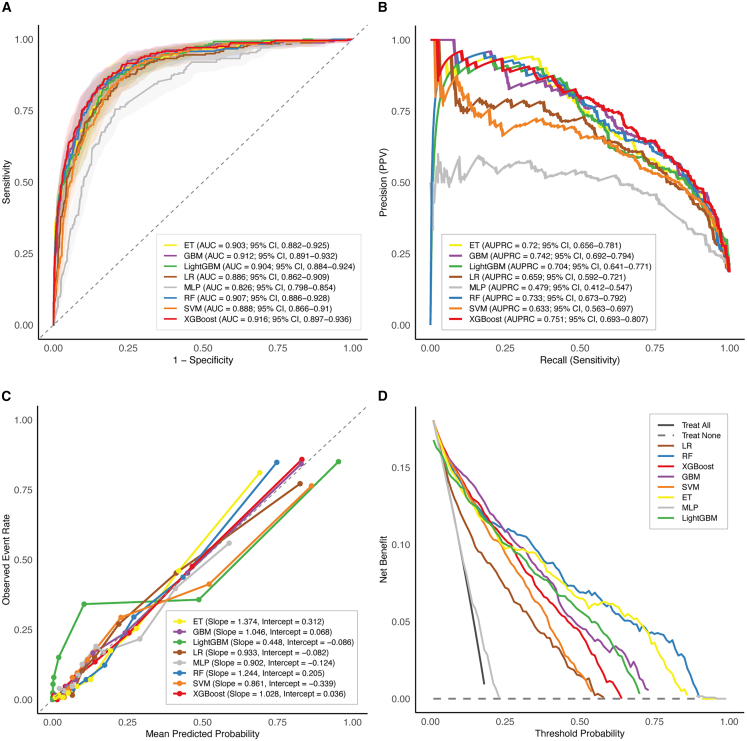
Performance evaluation of eight ML models in the training cohort (A) Discrimination assessed by ROC curves. Shaded areas represent the 95% confidence intervals (CIs). (B) PR curves for eight ML models. Precision represents the proportion of true positive predictions among all positive predictions, while recall reflects the proportion of true positives correctly identified among all actual positive cases. A higher AUPRC indicates better overall precision and recall. (C) Calibration plots evaluating agreement between mean predicted probability and observed event rate. Calibration performance was assessed using a decile-based calibration plot and evaluated by calibration intercept and slope. The dashed black line represents the ideal 45-degree reference line. Deviation above or below this line indicates overestimation or underestimation of risk, respectively. (D) DCA comparing net clinical benefits. The “treat all” curve assumes that all patients with SFTS receive intensive management regardless of predicted mortality risk, whereas the “treat none” line assumes that no patients receive intensive management.

The eleven LASSO-selected predictors were used to develop eight ML models, including logistic regression (LR), random forest (RF), XGBoost, gradient boosting machine (GBM), LightGBM, support vector machine (SVM), extremely randomized trees (ET), and multilayer perceptron (MLP). Model training and evaluation were conducted within the training cohort using 10-fold cross-validation. All eight models demonstrated satisfactory discrimination for predicting mortality risk among patients with SFTS, with AUCs ranging from 0.826 to 0.916 (Figure 2A). XGBoost achieved the highest AUC of 0.916 (95% CI, 0.897–0.936), followed closely by GBM (0.912; 95% CI, 0.891–0.932), RF (0.907; 95% CI, 0.886–0.928), LightGBM (0.904; 95% CI, 0.884–0.924), and ET (0.903; 95% CI, 0.882–0.925). SVM and LR yielded AUCs of 0.888 (95% CI, 0.866–0.910) and 0.886 (95% CI, 0.862–0.909), respectively, while MLP exhibited the lowest AUC of 0.826 (95% CI, 0.798–0.854). In addition, XGBoost demonstrated favorable diagnostic metrics, achieving a sensitivity of 0.810, a specificity of 0.864, an F1 score of 0.905, an accuracy of 0.854, a positive predictive value (PPV) of 0.578, a negative predictive value (NPV) of 0.952, and a Brier score of 0.104. Detailed performance metrics for all eight ML models—including the AUCs with 95% CIs, sensitivity, specificity, F1 score, accuracy, PPV, NPV, and Brier score—are summarized in Table S1. Pairwise DeLong tests were performed to statistically compare the AUCs among the eight ML models. The *p* values are presented in a heatmap (Figure S1).

As shown in Figure 2B, the area under the precision-recall curve (AUPRC) ranged from 0.633 to 0.751 across the eight ML models. XGBoost achieved the highest AUPRC of 0.751 (95% CI, 0.693–0.807), followed by GBM (0.742; 95% CI, 0.692–0.794), RF (0.733; 95% CI, 0.673–0.792), ET (0.720; 95% CI, 0.656–0.781), and LightGBM (0.704; 95% CI, 0.641–0.771). While LR, SVM, and MLP demonstrated lower AUPRCs. Across the range of predicted probabilities, all models exhibited reasonable calibration. XGBoost, GBM, RF, and ET showed the closest agreement between predicted and observed event rates, with calibration curves closely aligning with the ideal 45-degree reference line. While SVM, LR, LightGBM, and MLP displayed mild deviations. Among them, XGBoost exhibited the most balanced calibration, with a slope of 1.028 and an intercept of 0.036, closely approximating the ideal values of 1 and 0, respectively (Figure 2C). DCA for the eight ML models in the training cohort is presented in Figure 2D. Across a wide range of threshold probabilities, RF, ET, LightGBM, XGBoost, and GBM provided the highest net benefit compared with the “treat all” and “treat none” strategies. LR and SVM demonstrated relatively lower net benefits.

### Model validation in the temporal validation cohort

**Figure 3:**
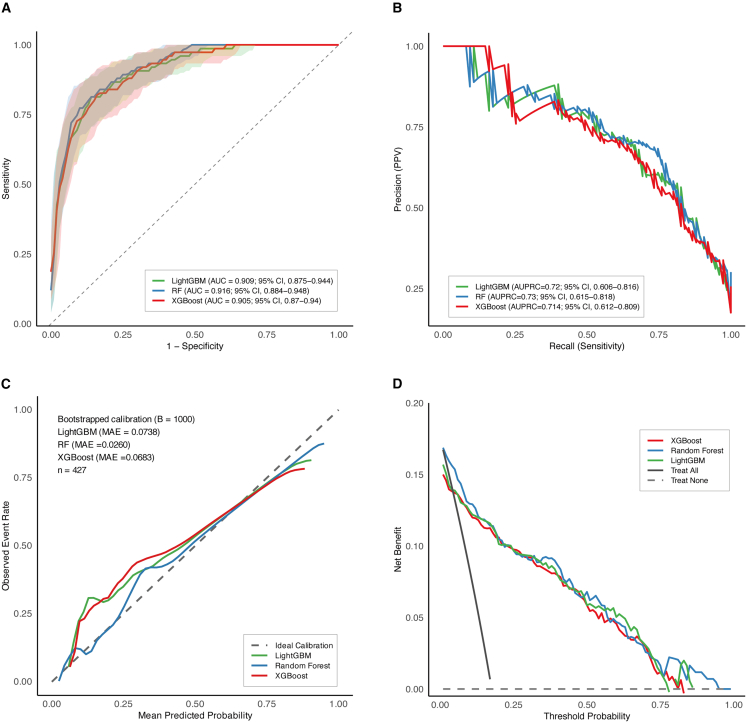
Validation of RF, LightGBM, and XGBoost models in the validation cohort (A) ROC curves of the three models in the validation cohort. Shaded areas represent the 95% confidence intervals (CIs). (B) PR curves of the three models in the validation cohort. (C) Calibration plots of the three models in the validation cohort. Bootstrapped calibration (B = 1,000 repetitions) was performed to adjust for optimism. The dashed line represents perfect calibration. (D) DCA of the three models in the validation cohort.

The top three models with the best performance in the training cohort—XGBoost, LightGBM, and RF—were further validated in the temporal validation cohort. The RF model achieved the highest AUC of 0.916 (95% CI, 0.884–0.948), followed by LightGBM (0.909; 95% CI, 0.875–0.944) and XGBoost (0.905; 95% CI, 0.870–0.940) (Figure 3A). Detailed performance metrics including AUC, 95% CI, sensitivity, specificity, F1 score, accuracy, PPV, NPV, and Brier score for all three models are summarized in Table S2. Pairwise DeLong tests were performed to statistically compare the AUCs among the eight ML models. The *p* values are presented in a heatmap (Figure S2). Consistent findings were observed in the PR curve analysis, where RF demonstrated the highest AUPRC (0.730; 95% CI, 0.615–0.818), followed by LightGBM (0.720; 95% CI, 0.606–0.816) and XGBoost (0.714; 95% CI, 0.612–0.809) (Figure 3B). Calibration plots indicated good agreement between mean predicted probabilities and observed event rate for all three models, with mean absolute errors (MAEs) of 0.026, 0.068, and 0.074 for RF, XGBoost, and LightGBM, respectively (Figure 3C). DCA exhibited that all three models provided higher net benefits across a wide range of threshold probabilities compared to “treat all” or “treat none” strategies (Figure 3D).

To further assess improvements in classification between models, NRI analyses were conducted. Compared with RF, both XGBoost (NRI = 0.230; 95% CI, 0.051–0.391; *p* = 0.008) and LightGBM (NRI = 0.200; 95% CI, 0.047–0.359; *p* = 0.010) showed significant improvements in reclassification. In contrast, no significant difference was observed between XGBoost and LightGBM (NRI = −0.035; 95% CI, −0.184–0.112; *p* = 0.630). IDI could not be reliably estimated for any of the model comparisons due to the highly similar predicted probabilities, resulting in negligible differences in discrimination slopes (Table S3).

### Model interpretation and feature importance

**Figure 4:**
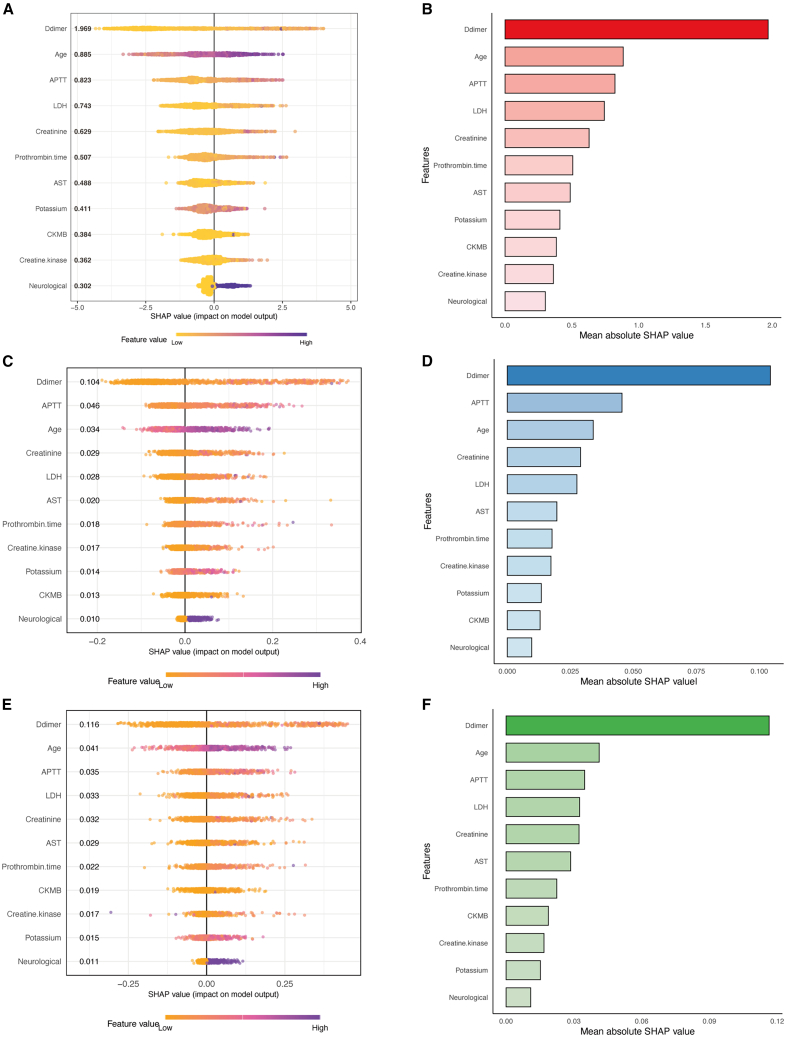
SHAP summary plot and feature importance of the models (A) SHAP summary plot for the XGBoost model. Higher SHAP values indicate a greater probability of mortality. Each dot represents an individual patient, with the color indicating the relative magnitude of the feature value and the position along the *x* axis denoting the SHAP value. (B) Feature importance rankings for the XGBoost model. (C) SHAP summary plot for the RF model. (D) Feature importance ranking for the RF model. (E) SHAP summary plot for the LightGBM model. (F) Feature importance ranking for the LightGBM model.

To enhance model interpretability and facilitate clinical application, SHAP summary plots and feature importance rankings were generated for the top-performing models: XGBoost, RF, and LightGBM. In the XGBoost model, D-dimer was identified as the most influential predictor for mortality, followed by age, APTT, LDH, and creatinine (Figures 4A and 4B). In the RF model, D-dimer, APTT, age, creatinine, and LDH consistently emerged as the leading contributors to mortality prediction (Figures 4C and 4D). Similarly, in the LightGBM model, D-dimer accounted for the highest relative importance, followed by age, APTT, LDH, and creatinine (Figures 4E and 4F).

Based on consistent feature rankings across models and considering of model simplicity and clinical applicability, six key variables (D-dimer, age, APTT, LDH, creatinine, and AST) were selected to develop a simplified XGBoost model with reduced feature set. In the training cohort, this model achieved an AUC of 0.910 (95% CI, 0.889–0.931) (Figure S3A), which was comparable to that of the original 11-variable model (AUC = 0.916; 95% CI, 0.897–0.936), indicating minimal loss of discriminative performance. In the temporal validation cohort, the six-variable XGBoost model yielded an AUC of 0.916 (95% CI, 0.888–0.945) (Figure S3B), demonstrating strong generalizability. Table S4 summarizes the performance metrics, including sensitivity, specificity, F1-score, accuracy, PPV, NPV, and Brier score in both the training and validation cohorts. We subsequently employed SHAP dependence plots to further illustrate how these top features affect the model output (Figure S4).

### Web-based implementation of a clinical prediction tool

To facilitate real-time clinical decision-making, we developed and deployed a publicly accessible web-based tool based on the six-variable XGBoost model to predict mortality risk in patients with SFTS. The platform is freely available at https://sftsprognosis.com, and it allows clinicians to input individual patient data and instantly obtain personalized risk estimates and stratification (Figure S5). In addition, the tool provides SHAP-based interpretability outputs, offering a detailed breakdown of how each predictor contributes to the overall risk, thereby enhancing transparency and guiding early clinical intervention strategies in patients with SFTS.

## Discussion

This multicenter retrospective cohort study is based on a decade of clinical data from 1,690 hospitalized SFTS patients across five hospitals in China. The aim was to develop and deploy a clinical practical early mortality prediction model using routinely available admission variables via an open-access web platform. Using LASSO regression for feature selection from 47 clinical parameters, we trained and compared eight supervised ML algorithms. Among them, XGBoost achieved the most favorable discriminative performance, with an AUC of 0.916 and AUPRC of 0.751 in the training cohort and an AUC of 0.905 and AUPRC of 0.714 in the temporal validation cohort. To improve interpretability and clinical applicability, SHAP was employed to explain the contribution of each variable to model predictions. Six key predictors (D-dimer, age, APTT, LDH, creatinine, and AST) emerged as the most influential and clinically relevant. These six variables were subsequently used to build an XGBoost model, which was deployed online to provide real-time, individualized mortality risk estimates with transparent SHAP-based visual outputs. The platform is designed to support early risk stratification and clinical decision-making, particularly in patients with rapidly deteriorating conditions. Our analytical framework was methodologically sound, incorporating a standardized pipeline encompassing data preprocessing, feature selection, model development, and validation.^24^^,^^25^ This study was conducted and reported in accordance with the recently published TRIPOD+AI guidelines to ensure transparency, reproducibility, and completeness in the development and evaluation of ML-based clinical prediction models.^26^

ML is increasingly being applied in clinical and therapeutic domains due to its ability to learn complex and nonlinear relationships from high-dimensional, heterogeneous data, surpassing the capabilities of traditional statistical methods.^27^ The COVID-19 pandemic has further underscored the value of ML in infectious disease modeling, with numerous studies demonstrating its superiority over conventional classification methods.^28^ ML algorithms have shown growing promise in infectious diseases, offering more scalable and timely solutions for diagnostic and clinical decision-making strategies.^29^ A recent systematic review identified XGBoost, RF, LR, and SVM as the most commonly adopted ML algorithms for potential clinical deployment, particularly in predictive modeling across diverse disease areas.^30^ XGBoost, an optimized implementation of gradient boosting decision trees, has been widely adopted for its high accuracy, robustness, and scalability in large, complex clinical datasets. Unlike traditional logistic regression, XGBoost effectively captures nonlinear relationships and aggregates multiple weak learners to improve generalization.^31^^,^^32^^,^^33^

By systematically evaluating discrimination, calibration and clinical utility metrics across eight supervised ML algorithms, we found that ensemble-based models demonstrated the most consistent performance, with XGBoost achieving the most favorable performance in both training and temporal validation cohorts. The reliable performance of XGBoost in temporal validation supports the generalizability of the model across different time periods. These findings align with growing evidence supporting ensemble-based ML in clinical prediction. ML is often described as a “black box,” where data enters and decisions emerge, but the processes between input and output remain opaque.^34^ This lack of transparency continues to pose a major barrier to clinical trust and deployment in real-world settings.^30^ To address this limitation, we incorporated SHAP to enhance model interpretability, enabling clinicians to visualize and understand the most important factors and their contribution to the prediction.^35^ These combined methodological strengths underscore the model’s robustness, interpretability, and translational relevance in facilitating early risk stratification for patients with SFTS.

Based on model explainability analysis, six clinical variables were ultimately selected to train the XGBoost model. These variables not only ranked among the most influential features in SHAP interpretation, but also represent clinically meaningful markers of coagulation dysfunction, organ injury, and disease severity in patients with SFTS. D-dimer ranked highest in SHAP-based feature importance, reflecting activation of fibrinolysis and underlying coagulopathy, which have been consistently associated with poor prognosis and DIC in severe cases.^36^ Prolonged APTT further supports the presence of coagulation pathway disruption, reinforcing the prognostic relevance of hemostatic abnormalities in SFTS patients.^37^ Advanced age, widely recognized as a predictor of adverse outcomes, may impair host defenses through age-related immune senescence and an increased burden of comorbid conditions.^38^^,^^39^ Consistent with prior clinical and experimental evidence, our model incorporated key biomarkers of hepatic and renal dysfunction, including LDH, AST, and creatinine. Gong et al. reported that elevated AST and LDH levels, indicative of hepatic dysfunction, were significantly associated with fatal SFTS outcomes.^40^ Lee et al. identified elevated serum creatinine as an independent mortality risk factor, suggesting the prognostic value of renal impairment.^41^ Moreover, immunohistochemistry and RNA scope *in situ* hybridization demonstrated substantial SFTSV replication in the liver and kidneys of both aged and young adult ferrets, providing experimental evidence that reinforces the clinical significance of hepatic and renal dysfunction in the pathogenesis of SFTS.^42^ The variables identified in our study are consistent with previous clinical evidence and provide additional mechanistic insight, thereby enhancing the interpretability and real-world prognostic validity of the model.

Most previously prognostic models for SFTS have relied on traditional statistical approaches such as logistic regression or risk scoring systems, often developed using limited sample sizes, single-center cohorts and lacking external validation.^43^^,^^44^ Although these studies have identified relevant clinical risk factors associated with adverse outcomes in patients with SFTS, their generalizability and ability to generate individualized risk estimates remain limited. To facilitate real-world application, we deployed the simplified XGBoost model as a publicly accessible user-friendly, and cost-free web-based tool (https://sftsprognosis.com) that provides real-time, individualized mortality risk estimates alongside intuitive, interpretable visual outputs. This approach aligns with recent recommendations advocating for the development of simplified yet interpretable models that are readily adoptable by frontline clinicians, thereby enhancing clinical utility.^45^ SFTS has been increasingly reported across a growing number of countries and regions, with a seasonal peak from April to October.^12^ However, clinical recognition of SFTS remains suboptimal in some settings, and reliable tools for early prognostic assessment are seldom available in routine practice. By enabling early identification of high-risk patients and stratification into low-, intermediate-, and high-risk categories, our tool facilitates prompt clinical intervention and more efficient allocation of critical care resources. Ultimately, this platform has the potential to enhance clinical decision-making, optimize resource utilization, and reduce adverse outcomes among patients with SFTS.‬‬‬‬‬‬‬‬‬‬‬

This study systematically integrates ML based mortality prediction, model interpretability, and open-access web deployment for patients with SFTS, using a large, real-world multicenter cohort. Among the evaluated models, XGBoost demonstrated the best performance and was deployed as a publicly accessible web-based tool to facilitate clinical application. This translational step from algorithm development to clinical implementation addresses a long-standing gap in SFTS research by providing a practical tool for early risk prediction, stratification, clinical decision-making. The resulting online prediction platform offers accessible, real-time support for frontline physicians, facilitating timely intervention and more efficient allocation of critical care resources in the management of SFTS.

### Limitations of the study

There are several limitations. First, although the model was developed and validated using a ten-year, multicentre cohort, and all data were retrospectively collected from five tertiary hospitals in eastern China. While temporal validation improved robustness over time, the findings may not fully generalize across other geographic regions, healthcare systems, or patient populations. Future studies should expand patient recruitment across different regions to conduct multi-regional external validation and further establish the model’s transferability. Second, the usability and clinical impact of the web-based prediction tool were not evaluated in real-world settings. Future studies should include prospective testing and clinician feedback to assess its practical utility and feasibility of integration into routine clinical workflows. Third, although we selected routinely available variables to enhance clinical applicability, some potentially relevant biomarkers (cytokine profiles and viral load dynamics) were not included due to data unavailability. Fourth, advanced age is a recognized risk factor for adverse outcomes in patients with SFTS. However, our study did not incorporate detailed information on chronic comorbidities in older patients, which may have influenced the observed association between age and mortality. Future research should also include a broader range of clinical and laboratory variables and consider standardized comorbidity indices to more clearly elucidate the independent prognostic impact of age.

## Resource availability

### Lead contact

Requests for further information and resources should be directed to and will be fulfilled by the lead contact, Zhihai Chen (chenzhihai0001@126.com).

### Materials availability

This study did not generate new unique reagents.

### Data and code availability

The individual-level clinical and demographic patient data collected from the participating hospitals are not publicly available due to institutional regulations and ethics approval requirements. Access to these data may be granted upon reasonable request to the lead contact, subject to approval by the relevant institutional ethics committees and applicable data-sharing agreements. All original code has been deposited at https://github.com/chenmedoc/SFTS-Mortality-Risk-Prediction and is publicly available as of the date of publication.

Additional information required to reanalyze the data reported in this study is available from the lead contact upon request.

## Acknowledgments

The graphical abstract was created using BioRender.com. This work was supported by the 10.13039/501100012166National Key Research and Development Program of China (2022YFF1203201), the 10.13039/501100001809National Natural Science Foundation of China (82072295), and Beijing Research Center for Respiratory Infectious Diseases (BJRID2024-009).

## Author contributions

Conceptualization, Z.C., C.Z., T.Z., and Z.G.; data collection, D.T., Y.S., J.W., J.L., and Y.X.; data management, L.L., Z.Z., and J.D.; methodology, Z.C., C.Z., and T.Z.; machine learning, C.Z. and Z.G.; original draft preparation, C.Z., T.Z., and Z.G.; review and editing, Z.C., J.D., and L.L.; funding acquisition, Z.C.; supervision, Z.C. and C.Z. All authors read and approved the final manuscript.

## Declaration of interests

The authors declare no competing interests.

## STAR★Methods

### Key resources table

**Table:** 

REAGENT or RESOURCE	SOURCE	IDENTIFIER
**Software and algorithms**		

R software (v4.4.2)	R CRAN	https://cran.r-project.org/
glmnet R package (v4.1-10)	R CRAN	https://cran.r-project.org/web/packages/glmnet/index.html
dplyr R package (v1.1.4)	R CRAN	https://cran.r-project.org/web/packages/dplyr/index.html
caret R package (v7.0-1)	R CRAN	https://cran.r-project.org/web/packages/caret/index.html
xgboost R package (v1.7.11.1)	R CRAN	https://cran.r-project.org/web/packages/xgboost/index.html
lightgbm R package (v4.6.0)	R CRAN	https://cran.r-project.org/web/packages/lightgbm/index.html
pROC R package (v1.19.0.1)	R CRAN	https://cran.r-project.org/web/packages/pROC/index.html
ggplot2 R package (v4.0.0)	R CRAN	https://cran.r-project.org/web/packages/ggplot2/index.html
rms R package (v8.0-0)	R CRAN	https://cran.r-project.org/web/packages/rms/index.html
rmda R package (v1.6)	R CRAN	https://cran.r-project.org/web/packages/rmda/index.html
SHAPforxgboost R package (v0.1.3)	R CRAN	https://cran.r-project.org/web/packages/SHAPforxgboost/index.html
tidyr R package (v1.3.1)	R CRAN	https://cran.r-project.org/web/packages/tidyr/index.html
Codes for the model	Github	https://github.com/chenmedoc/SFTS-Mortality-Risk-Prediction
Python (v3.10)	Python Software Foundation	https://www.python.org
Streamlit (v1.29)	Streamlit Inc.	https://streamlit.io/

### Experimental model and study participant details

This retrospective multicentre cohort study included patients diagnosed with SFTS between January 2014 and December 2023 at five hospitals in China: Beijing Ditan Hospital, Yantai Qishan Hospital, Qingdao Sixth People’s Hospital, Dandong Infectious Disease Hospital, and Tai’an Central Hospital. Inclusion criteria were: (1) Age ≥18 years; (2) confirmed SFTS diagnosis based on detection of SFTSV by reverse transcription polymerase chain reaction (RT-PCR); (3) availability of key clinical and laboratory data within 48 h of admission. Exclusion criteria included: (1) patients with acute or chronic viral infections; (2) a history of hematologic disorders, including leukemia, platelet disorders, or coagulation abnormalities; (3) patients with malignant tumors undergoing antitumor therapy; (4) missing clinical data exceeding 10%.

A total of 1,690 patients were enrolled, including 312 non-survivors and 1378 survivors, classified based on clinical outcomes. The model was designed as a supervised binary classification task, aiming to distinguish between SFTS survivors and non-survivors based on clinical features. Patients admitted between January 2014 and December 2021 (*n* = 1263) were used as the training cohort, patients admitted between January 2022 and December 2023 (*n* = 427) formed the temporal validation cohort. This study was conducted and reported in accordance with the Transparent Reporting of a multivariable prediction model for Individual Prognosis Or Diagnosis (TRIPOD) +AI guidelines.^26^

This study protocol adhered to the principles of the Declaration of Helsinki and was approved by the Ethics Committee of Beijing Ditan Hospital (NO.DTEC-KY2022-022), Capital Medical University. As a retrospective study utilizing anonymized data, the requirement for informed patient consent was waived.

### Method details

#### Data collection and definition

Baseline demographics and clinical parameters were retrospectively extracted from electronic medical records within 48 h of hospital admission at each participating center, using a standardized data collection template. A total of 47 clinical variables were included in the analysis. Clinical symptoms and signs included temperature, chill, fatigue, headache, myalgia, arthralgia, inappetence, nausea, vomiting, diarrhea, neurological abnormalities, and lymphadenopathy, among others. Laboratory test parameters included white blood cell (WBC) count, neutrophils, lymphocytes, monocytes, red blood cell (RBC), hemoglobin, platelet count, mean platelet volume (MPV), serum potassium (K^+^), serum sodium (Na^+^), creatinine, blood urea nitrogen (BUN), lactate dehydrogenase (LDH), creatine kinase (CK), creatine kinase–MB isoenzyme (CK-MB), C-reactive protein (CRP), prothrombin time (PT), activated partial thromboplastin time (APTT), thrombin time (TT), D-dimer, alanine aminotransferase (ALT), aspartate aminotransferase (AST), total bilirubin (TBiL), direct bilirubin (DBiL), gamma-glutamyl transferase (GGT), albumin, globulin, cholinesterase, alkaline phosphatase (ALP), and total bile acid (TBA), among others.

The primary outcome, referred to as early mortality, was defined as in-hospital death attributable to SFTSV infection, including patients who died due to disease progression during hospitalization and those who experienced progressive clinical deterioration and subsequently withdrew from treatment.

Neurological abnormalities were defined as altered mental status (impaired consciousness, lethargy, or personality changes) lasting ≥24 h without an identifiable cause, or generalized/partial seizures not fully explained by a preexisting seizure disorder.^46^

#### Feature selection

To address missing data, continuous variables were imputed with the median and categorical variables with the mode, as the overall proportion of missing values was low (<10%). Prior to model training, continuous variables were standardized using *Z* score normalization within the training cohort. Feature selection was then performed using the least absolute shrinkage and selection operator (LASSO) regression, a regularization method that shrinks the coefficients of less informative predictors to exactly zero.^47^ To identify the optimal penalty parameter (λ), we applied 10-fold cross-validation within the training cohort, and retained predictors with non-zero coefficients at λ.1SE (standard error) threshold.^48^

#### Machine learning models

Based on the predictors selected via LASSO regression, eight supervised ML algorithms were developed to predict adverse outcomes in SFTS patients, including logistic regression (LR), random forest (RF), eXtreme Gradient Boosting (XGBoost), support vector machine (SVM), gradient boosting machine (GBM), Light Gradient Boosting Machine (LightGBM), extremely randomized trees (ET), and multilayer perceptron (MLP). To reduce overfitting and ensure robust performance, all models were trained using stratified 10-fold cross-validation with a fixed random seed for reproducibility. For most algorithms, hyperparameters were tuned via grid search using the caret package’s default tuning grids, and model performance was evaluated by the area under the receiver operating characteristic curve (AUC) computed with the twoClassSummary function. The optimal configuration for each model was selected based on mean cross-validated AUC, and out-of-fold predictions were retained for subsequent performance evaluation and calibration analysis. The XGBoost model was trained using caret’s xgbTree method, with hyperparameters tuned over key parameters, including the number of boosting rounds, tree depth, learning rate, and sampling ratios. The ET model was trained with a fixed configuration: the splitting rule was set to “extratrees”, mtry (number of features randomly selected at each split) was set to the square root of the number of predictors, and the minimum node size was fixed at 5. LightGBM was implemented with 100 boosting rounds and default values for learning and regularization parameters.

Model performance was evaluated in both the training and temporal validation cohorts using the AUC, 95% confidence interval (CI), sensitivity, specificity, precision, F1 score, positive predictive value (PPV), negative predictive value (NPV), and Brier score. DeLong test was employed to assess the statistical significance of differences in the AUCs between models. Precision-recall (PR) curves were utilized to evaluate the discriminative ability of the ML models, the area under the precision-recall curve (AUPRC) are widely recognized as a general measure of classifier performance.^49^ Calibration curves were constructed to assess the concordance between predicted probabilities and observed outcomes. Decision curve analysis (DCA) was performed to evaluate the clinical utility of each model by quantifying the net benefit across a range of threshold probabilities. Net Reclassification Improvement (NRI) and Integrated Discrimination Improvement (IDI) were calculated using the nricens package based on 1000 bootstrap resamples.^50^

#### Model interpretability and web-based tool deployment

SHAP analysis was conducted on the top-performing models to quantify feature contributions and improve model transparency and interpretability.^35^ For each model, an SHAP summary plot was generated to visualize the overall impact, importance, and directionality of individual features. Features importance was ranked based on their mean absolute SHAP values. In addition, dependence plots were performed for the top influential variables to further explore the relationship between feature values and predicted risk.

We trained the predictive model in R with XGBoost and exported it for use in Python, where SHAP was applied for interpretability. A web-based application was then implemented using the Streamlit framework, providing a free real-time tool that is publicly accessible for research demonstration.

### Quantification and statistical analysis

All statistical analyses, ML modeling, and visualizations were performed using R software (version 4.4.2). Key packages used included glmnet, dplyr, caret, xgboost, lightgbm, pROC, ggplot2, rms, rmda, SHAPforxgboost, tidyr, and others as appropriate. Categorical variables were presented as frequencies (n) and percentages (%) and compared using the Chi-square test. Continuous variables with a normal distribution were expressed as mean ± standard deviation and compared using the independent samples *t* test. Non-normally distributed continuous variables were reported as median and interquartile range (IQR), then analyzed using the Mann–Whitney U test. Statistical significance was defined as a two-tailed *p* value <0.05.
