# Supplementary material for: Machine learning models for early mortality prediction in severe fever with thrombocytopenia syndrome
Source: iScience. 2026 Jan 29;29(3):114843. doi: 10.1016/j.isci.2026.114843 (PMC12936484; doi:10.1016/j.isci.2026.114843)
Supplement: Document S1. Figures S1–S5 and Tables S1–S4 [file mmc1.pdf]

iScience, Volume *29*

Supplemental information

Machine learning models for early mortality prediction in severe fever with thrombocytopenia syndrome

Chenxi Zhao, Tingyu Zhang, Ziruo Ge, Ling Lin, Di Tian, Yi Shen, Zhenghua Zhao, Jingxia Wang, Jianming Lai, Yanli Xu, Jianping Duan, and Zhihai Chen

Table S1. Performance of machine learning models in the training cohort, related to Figure 2.

	LightGBM	LR	RF	XGBoost	SVM	GBM	ET	MLP
AUC	0.904	0.886	0.907	0.916	0.888	0.912	0.903	0.826
95%CI (AUC)	0.884-0.924	0.862-0.909	0.886-0.928	0.897-0.936	0.866-0.910	0.891-0.932	0.882-0.925	0.798-0.854
Sensitivity	0.549	0.781	0.565	0.810	0.793	0.823	0.570	0.755
Specificity	0.950	0.843	0.947	0.864	0.830	0.857	0.942	0.792
F1 score	0.925	0.890	0.925	0.905	0.884	0.903	0.923	0.857
Brier score	0.100	0.129	0.087	0.104	0.126	0.110	0.091	0.167
Accuracy	0.875	0.831	0.876	0.854	0.823	0.850	0.785	0.873
PPV	0.718	0.535	0.713	0.578	0.519	0.570	0.696	0.457
NPV	0.901	0.943	0.904	0.952	0.946	0.954	0.905	0.933
AUPRC	0.704	0.659	0.733	0.751	0.633	0.742	0.720	0.479
95%CI (AUPRC)	0.641-0.771	0.592-0.721	0.673-0.792	0.693-0.807	0.563-0.697	0.692-0.794	0.656-0.781	0.412-0.547
Calibration slope	0.448	0.933	1.244	1.028	0.861	1.046	1.374	0.902
Intercept	-0.086	-0.082	0.205	0.036	-0.339	0.068	0.312	-0.124

Notes: *AUC* area under the receiver operating characteristic curve, *CI* confidence interval, *PPV* positive predictive value, *NPV* negative predictive value, *LightGBM* Light gradient boosting machine, *LR* logistic regression, *RF* random forest, *XGBoost* eXtreme Gradient Boosting, *SVM* support vector machine, *GBM* gradient boosting machine, *ET* extra tree, *MLP* multilayer perceptron.

Table S2. Performance of machine learning models in the validation cohort, related to Figure 3.

	LightGBM	XGBoost	RF
AUC	0.909	0.905	0.916
95% CI	0.875-0.944	0.870-0.940	0.884-0.948
Sensitivity	0.547	0.573	0.547
Specificity	0.955	0.949	0.966
F1 score	0.931	0.930	0.937
Brier score	0.087	0.090	0.083
Accuracy	0.883	0.883	0.892
PPV	0.719	0.705	0.774
NPV	0.908	0.913	0.909

Notes: *AUC* area under the receiver operating characteristic curve, *CI* confidence interval, *PPV* positive predictive value, *NPV* negative predictive value, *LightGBM* Light gradient boosting machine, *XGBoost* eXtreme Gradient Boosting, *RF* random forest.

Table S3. NRI and IDI for model comparisons in the validation cohort, related to Figure 3.

	XGBoost vs LightGBM	XGBoost vs RF	LightGBM vs RF
NRI estimate	-0.035	0.230	0.200
NRI standard error	0.073	0.087	0.078
NRI 95%CI	-0.184 ~ 0.112	0.051-0.391	0.047-0.359
NRI z value	-0.482	2.653	2.559
NRI *P* value	0.630	0.008	0.010
IDI *P* value	Not estimable	Not estimable	Not estimable

Notes: NRI and IDI estimates are shown with 95% CIs and *P* values based on 1000 bootstrap replications. For comparisons in which IDI could not be reliably estimated, results are reported as "Not estimable". The statistical significance of NRI was assessed using Z statistics, calculated as the ratio of the point estimate to its standard error, with *P* values were derived from the standard normal distribution. *NRI* Net Reclassification Improvement, *IDI* Integrated Discrimination Improvement, *CI* confidence interval, *LightGBM* Light gradient boosting machine, *XGBoost* eXtreme Gradient Boosting, *RF* random forest.

Table S4. Performance metrics of the XGBoost model trained with six predictors in the training and validation cohorts.

	Training cohort	Validation cohort
AUC	0.910	0.916
95% CI	0.889-0.931	0.888-0.945
Sensitivity	0.802	0.827
Specificity	0.859	0.847
F1 score	0.664	0.649
Brier score	0.109	0.111
Accuracy	0.848	0.843
PPV	0.567	0.534
NPV	0.949	0.958

Notes: *AUC* area under the receiver operating characteristic curve, *CI* confidence interval, *PPV* positive predictive value, *NPV* negative predictive value, *XGBoost* eXtreme Gradient Boosting.

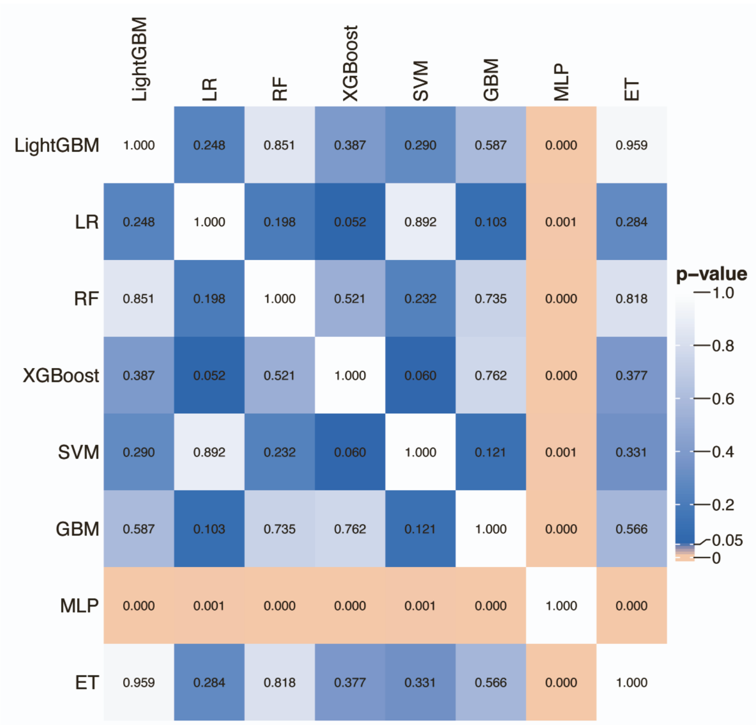

Figure S1. The statistical comparison between models was performed using DeLong test with Bonferroni correction in training cohort, related to Figure 2.

ET extremely randomized trees, *GBM* gradient boosting machine, *LightGBM* Light Gradient Boosting Machine, *LR* logistic regression, *MLP* multilayer perceptron, *RF* random forest, *SVM* support vector machine, *XGBoost* eXtreme Gradient Boosting.

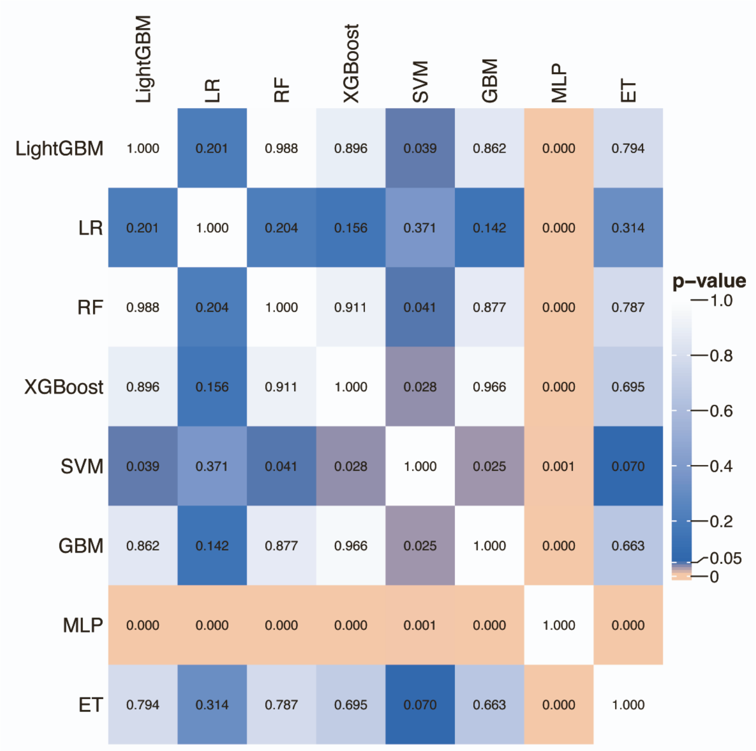

Figure S2. The statistical comparison between models was performed using DeLong test with Bonferroni correction in validation cohort, related to Figure 3.

ET extremely randomized trees, *GBM* gradient boosting machine, *LightGBM* Light Gradient Boosting Machine, *LR* logistic regression, *MLP* multilayer perceptron, *RF* random forest, *SVM* support vector machine, *XGBoost* eXtreme Gradient Boosting.

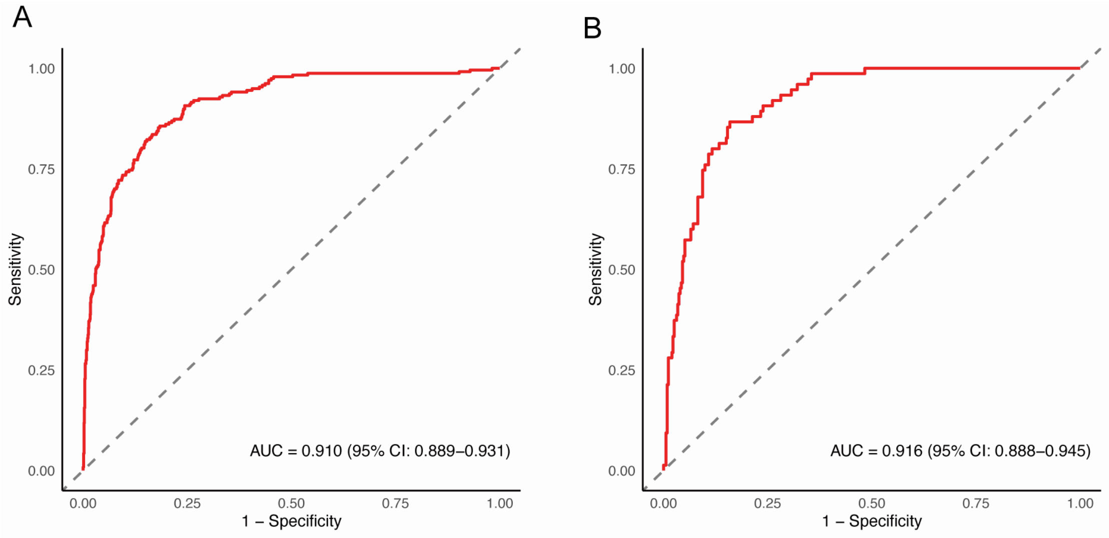

Figure S3. Performance of XGBoost model trained with six features in the training and validation cohorts.

(A) ROC curve of the XGBoost model in the training cohort. (B) ROC curve of the XGBoost model in the validation cohort. *AUC* area under the receiver operating characteristic curve, *CI* confidence interval, *ROC* receiver operating characteristic.

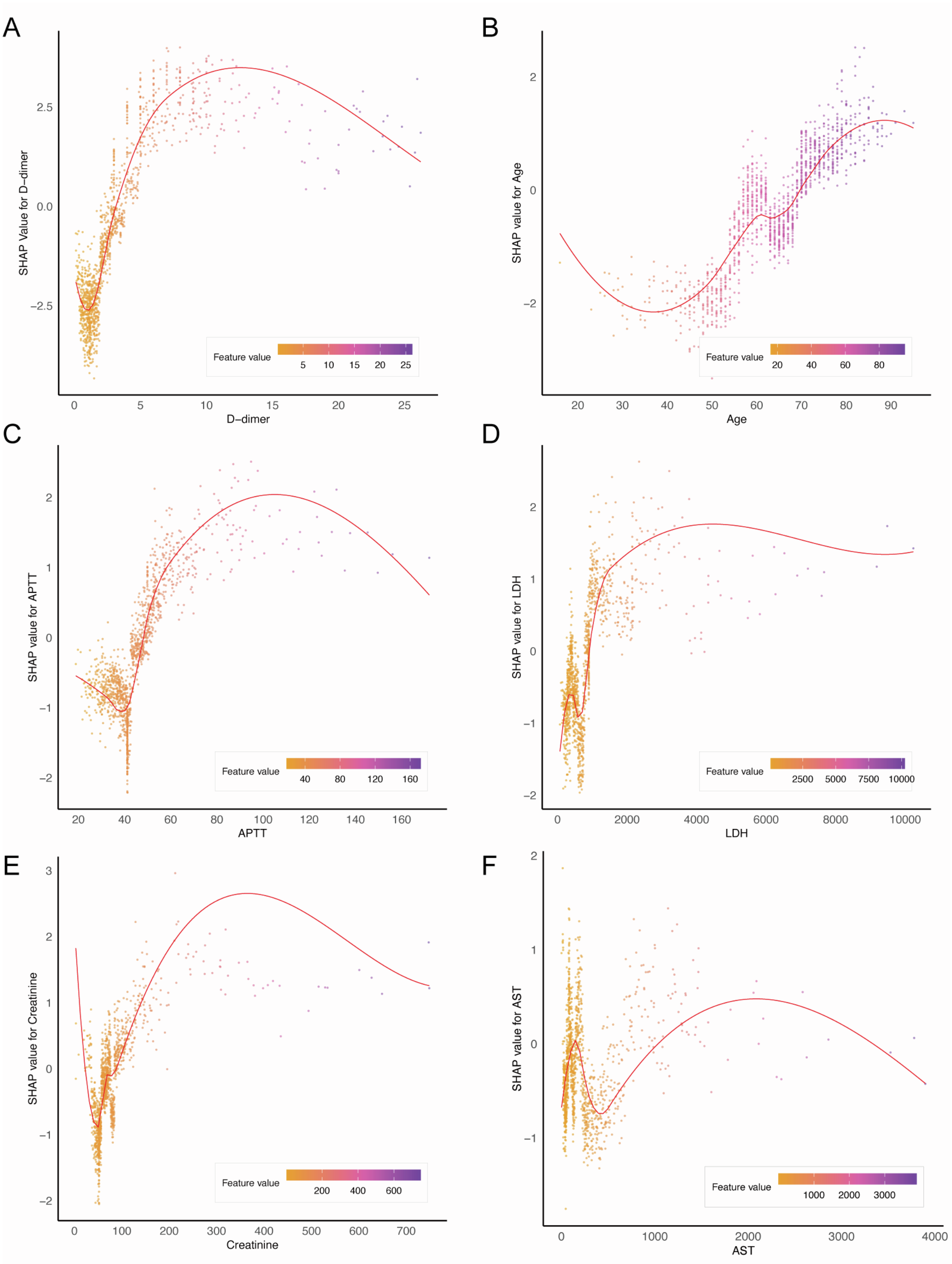

Figure S4. SHAP dependence plots for the top six predictors in the XGBoost model, related to Figure 4.

(A) D-dimer, (B) Age, (C) APTT, (D) LDH, (E) Creatinine, (F) AST. Higher SHAP values indicate a greater contribution of the feature toward predicting mortality. Each dot represents one patient, with the color gradient indicating the magnitude of the feature value. The red smoothed line represents the general trend of feature impact across the range of feature values. *SHAP* SHapley Additive exPlanations, *XGBoost* eXtreme Gradient Boosting, *APTT* activated partial thromboplastin time, *LDH* lactate dehydrogenase, *AST* aspartate aminotransferase.

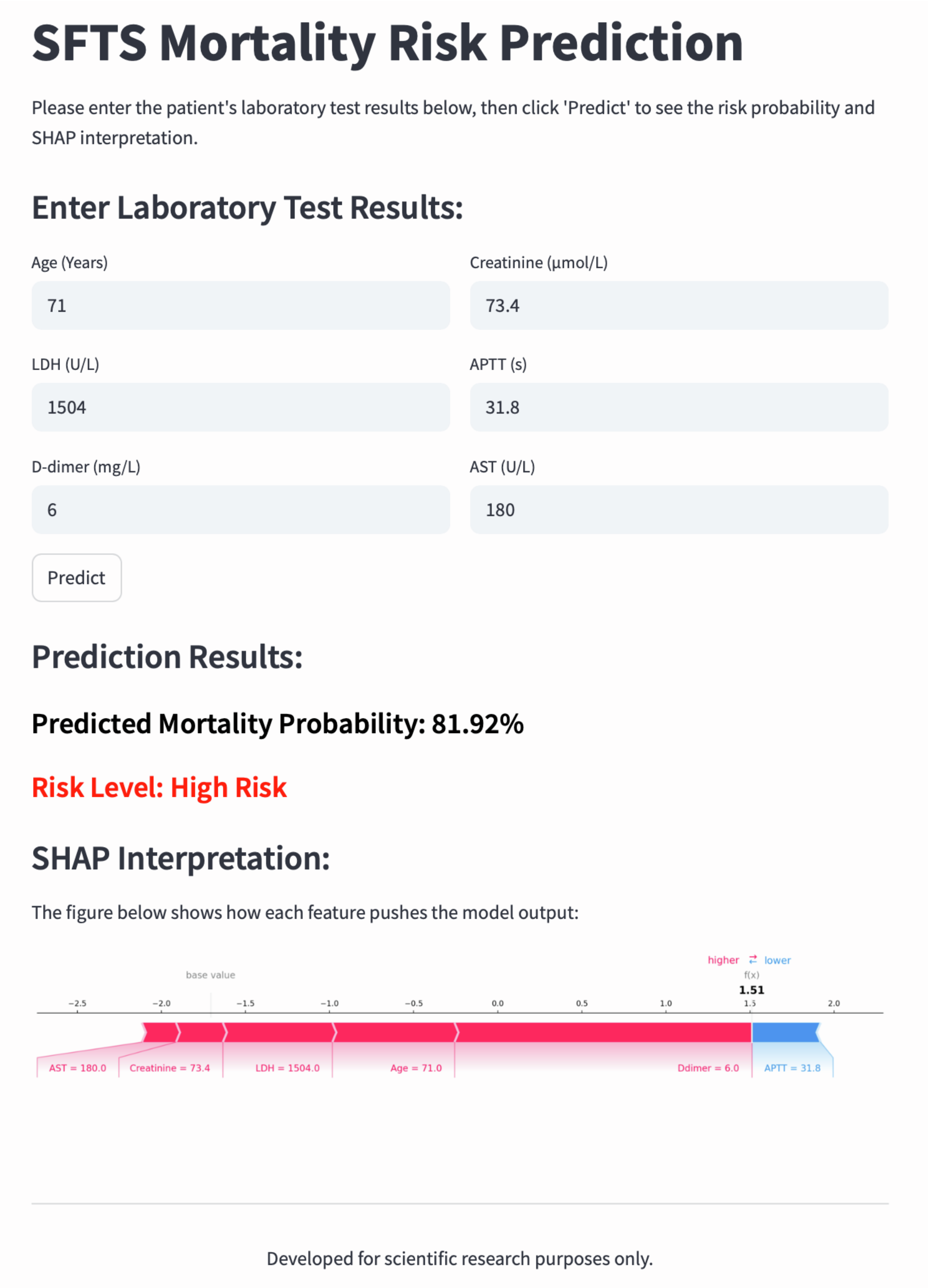

Figure S5. Screenshot of the web-based SFTS mortality risk prediction tool.

The interface allows users to enter six routinely available clinical variables (Age, Creatinine, LDH, APTT, D-dimer, AST). For demonstration, one patient's case data is shown. The output provides the predicted mortality probability, stratified risk level, and a SHAP-based interpretation plot indicating the contribution of each variable to the prediction. The footer states that the tool is developed for research purposes only.
